# Regulatory roles of G-protein coupled receptors in adipose tissue metabolism and their therapeutic potential

**DOI:** 10.1007/s12272-021-01314-w

**Published:** 2021-02-07

**Authors:** Hyeonyeong Im, Ji-Hyun Park, Seowoo Im, Juhyeong Han, Kyungmin Kim, Yun-Hee Lee

**Affiliations:** College of Pharmacy and Research Institute of Pharmaceutical Sciences, Seoul National University; Bio-MAX Institute, Seoul National University, 29-Room # 311, 1 Gwanak-ro, Gwanak-gu, Seoul, 08826 Republic of Korea

**Keywords:** GPCR, Obesity, Adipose tissue, Adrenoceptor, Adenosine receptor, Frizzled receptor, Lysophospholipid receptor

## Abstract

The high incidence of obesity has increased the need to discover new therapeutic targets to combat obesity and obesity-related metabolic diseases. Obesity is defined as an abnormal accumulation of adipose tissue, which is one of the major metabolic organs that regulate energy homeostasis. However, there are currently no approved anti-obesity therapeutics that directly target adipose tissue metabolism. With recent advances in the understanding of adipose tissue biology, molecular mechanisms involved in brown adipose tissue expansion and metabolic activation have been investigated as potential therapeutic targets to increase energy expenditure. This review focuses on G-protein coupled receptors (GPCRs) as they are the most successful class of druggable targets in human diseases and have an important role in regulating adipose tissue metabolism. We summarize recent findings on the major GPCR classes that regulate thermogenesis and mitochondrial metabolism in adipose tissue. Improved understanding of GPCR signaling pathways that regulate these processes could facilitate the development of novel pharmacological approaches to treat obesity and related metabolic disorders.

## Introduction

G protein-coupled receptors (GPCRs), one of the largest protein receptor families, are seven-transmembrane-domain protein receptors that mediate various downstream signals by interacting with G protein complexes (α and βγ) (Weis and Kobilka 2018).

Ligand binding to GPCRs induces conformational changes in the receptors, which activates exchange of guanosine triphosphate for guanosine diphosphate on the Gα subunits (Marinissen and Silvio Gutkind 2001), resulting in the dissociation of the Gβγ dimer from Gα. Based on their sequence homologies and functional similarities, Gα proteins are categorized into four major groups: Gα_s_, Gα_i/o_, Gα_q_, and Gα_12/13_ (Syrovatkina et al. 2016), and activate distinct downstream effectors (Syrovatkina et al. 2016).

GPCRs are the most successful class of druggable targets in human diseases (Quiñones et al. 2019), estimated to be targeted by approximately 34% of the FDA-approved drugs (Hauser et al. 2017). The disease indications for GPCR regulators, including cardiovascular, immunological, and metabolic disorders, have been greatly expanded (Hauser et al. 2017). For example, glucagon-like peptide-1 receptor agonists for the treatment of obesity and type 2 diabetes mellitus are a well-known class of recently approved drugs that act on GPCRs (Husted et al. 2017; Sloop et al. 2018).

Adipose tissue, which can be white or brown, is a major metabolic organ that regulates energy homeostasis (Kershaw and Flier 2004; Cannon and Nedergaard 2004). Adipocytes are specialized cell types responsible for lipid metabolism, including catabolic lipolysis, anabolic *de novo* lipogenesis, and triglyceride (TG) accumulation (Rosen and Spiegelman 2006). In general, white adipose tissue (WAT) is an endocrine organ that stores surplus nutrients as TG in lipid droplets, occupying 95% of the cell mass (Trayhurn and Beattie 2001; Lee, Wu, and Fried 2013). During negative energy balance, WAT hydrolyzes TG and mobilizes free fatty acids (FFA) into the circulation to supply systemic energy demands (Rosen and Spiegelman 2014).

Continuous positive energy balance results in hypertrophic expansion of WAT and TG accumulation exceeding the storage capacity of adipose tissue, causing ectopic fat accumulation in non-adipose organs (Sun et al. 2013; Rosen and Spiegelman 2006). Moreover, this hypertrophic response is often associated with inflammation, insulin resistance, and metabolic syndromes (Verboven et al. 2018; Grundy 2004; Furukawa et al. 2017). Although lipolysis can be targeted to reduce fat mass, excess WAT lipolysis is associated with increased circulating FFAs and lipotoxicity, which might contribute to nonalcoholic fatty liver disease and insulin resistance (Samuel and Shulman 2012). Therefore, pharmacological targeting of WAT lipolysis might be challenging due to adverse effects.

In contrast, brown adipose tissue (BAT) is a specialized thermoregulatory organ that dissipates FFAs into heat (Cannon and Nedergaard 2004). BAT contains high levels of mitochondria and is molecularly characterized by uncoupling protein 1 (UCP1) expression (Cannon and Nedergaard 2004). It has been well accepted that activation of BAT metabolism results in enhanced energy expenditure and improved insulin sensitivity and metabolic profiles (Peng et al. 2015; Kajimura, Spiegelman, and Seale 2015). Importantly, white adipocytes can be converted into brown like adipocytes under physiological and pharmacological thermogenic stimuli, a phenomenon defined as WAT browning (Lee, Mottillo, and Granneman 2014; Kajimura, Spiegelman, and Seale 2015). Therefore, molecules that regulate the thermogenic pathways in BAT and WAT have been intensively studied as a promising target to treat or prevent obesity and obesity-related metabolic syndromes (Kim and Plutzky 2016).

In this review, we summarized recent findings on five GPCR subfamilies that regulate thermogenesis and mitochondrial metabolism in BAT and WAT: beta adrenoceptors, alpha adrenoceptors, adenosine receptors, frizzled receptors, and lysophospholipid receptors. Our goal is to provide the current understanding of GPCR-related molecular mechanisms that regulate adipose tissue metabolism and potentially aid the development of novel targets for obesity and obesity-related metabolic diseases.

### Relative abundance of gene expression levels of GPCRs in adipose tissue

We analyzed and compared the relative expression levels of GPCRs in mice and humans using in-house and public RNAseq data (GSE148275 and GSE135134). The list of genes classified into GPCRs was obtained from the IUPHAR/BPS guide to pharmacology (http://www.guidetopharmacology.org/GRAC/FamilyDisplayForward?familyId=694).

**Fig. 1:**
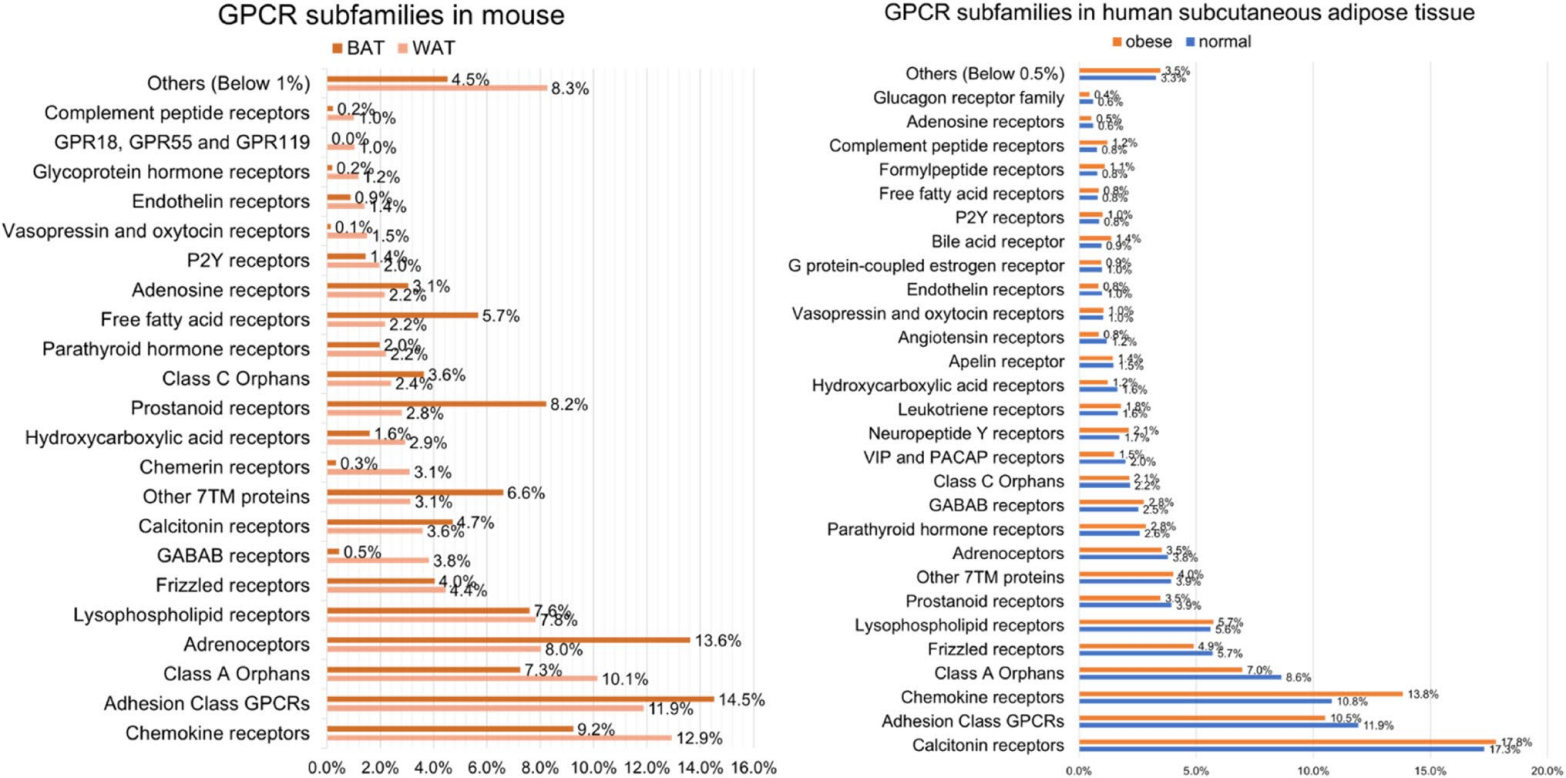
Relative expression levels of GPCR subfamilies expressed in mouse and human adipose tissue (GSE148275 and GSE135134). The relative expression levels of GPCRs in mouse and human adipose tissue are presented based on public transcriptome data (GSE148275 and GSE135134). Wild type mice analyzed in GSE138275 were treated with tamoxifen, and analysis was performed after a wash-out period (2 weeks). Using the GEO dataset GSE135134, we separated the group into two, and obese includes individuals with BMI >  = 30 kg/m^2^, normal with BMI < 25 kg/m^2^. A list of mus musculus and homo sapiens GPCR genes was referred to IUPHAR/BPS guide to pharmacology (http://www.guidetopharmacology.org/GRAC/FamilyDisplayForward?familyId=694). We grouped the subfamilies below 1% (or 0.5%) of total GPCRs in the title of Others (Below 1% (or 0.5%))

GPCR subfamilies expressed higher than 1% of the total GPCR expression in mouse WAT are listed and compared to the distribution of GPCR subfamilies in mouse BAT in Fig. 1. Similarly, GPCR subfamilies expressed higher than 0.5% of the total GPCR expression in human subcutaneous WAT from individuals with normal adiposity (body mass index (BMI) < 25) are listed and compared to the distribution of GPCR subfamilies in WAT of obese individuals (BMI ≥ 30).

Based on previous studies related to the regulation of thermogenesis and mitochondrial metabolism in adipose tissue, we reviewed five GPCR subfamilies, namely beta adrenoceptors, alpha adrenoceptors, adenosine receptors, frizzled receptors, and lysophospholipid receptors. G protein subtypes coupling to the specific GPCRs are listed in Table 1, and the effects of the GPCR agonists/antagonists on adipose tissue metabolism are summarized in Table 2.

**Table 1:** GPCR with its coupling G-proteins

Receptors	Subtypes	Coupling G-protein	References
Beta adrenoceptors	β1-AR	Gαs	Gurdal, Friedman, and Johnson (1995)
	β2-AR		
	β3-AR		
Alpha adrenoceptors	α1-AR	Gαo	Evans et al. (2019)
	α2-AR	Gαi	
Adenosine receptors	A_1_R	Gαi	Tozzi and Novak (2017b)
	A_2A_	Gαs	
	A_2B_	Gαs	
	A_3_R	Gαi	
Frizzled receptors	FZD1	Gαi, Gαq, Gαs, Gα12/13	Nichols et al. (2013), Park et al. (2015), Schulte and Wright (2018)
	FZD2	Gαi, Gαq	
	FZD3	Gαs	
	FZD4	Gαi, Gα12/13	
	FZD5	Gαq	
	FZD6	Gαs, Gαq, Gαi	
	FZD7	Gαs, Gαq, Gαi	
	FZD8	N.D	
	FZD9	Gαo	
	FZD10	Gαi/o,Gα13	
Lysophospholipid receptors	LPA1	Gαi, Gαq, Gα12/13	Rosen et al. (2009)
	LPA2	Gαi, Gαq, Gα12/13	
	LPA3	Gαi, Gαq	
	LPA4	Gα12/13	Yanagida et al. (2018)
	LPA5	Gαq, Gα12/13	Yung, Stoddard, and Chun (2014)
	LPA6	Gα12/13	
	S1PR1	Gαi	Rosen et al. (2009)
	S1PR2	Gαs, Gαq, Gα12/13	
	S1PR3	Gαi, Gα12/13	
	S1PR4		
	S1PR5

**Table 2:** GPCR agonists/antagonists that regulate adipose tissue metabolism

Subfamilies		Pharmacological agents	Effect	References
β Adrenoceptors	Non-selective	Isoproterenol (agonist)	Increased mitochondrial activity in 3T3-L1 adipocytes	Cho et al. (2009)
			UCP1 induction in human adipocytes	Evans et al. (2019), Riis-Vestergaard, Richelsen et al. (2020)
	β1	Dobutamine (agonist)	Increased lipolysis and thermogenesis in human, UCP1 induction in human brown adipocytes	Green et al. (1992), Riis-﻿Vestergaard﻿, Richelsen et al. (2020)
		Talinolol (antagonist)	Reduced browning of cold exposure in mice	Jiang et al. (2017)
	β2	Terbutaline (agonist)	Increased lipolysis in human	Enocksson et al. (1995)
	β3	CL316,243 (agonists)	Increased thermogenesis, energy expenditure and BAT hypertrophy in rats	Himms-Hagen et al. (1994)
		Mirabegron (agonists)	BAT thermogenesis, WAT lipolysis, and weight loss in humans	Cypess, Weiner et al. (2015)
α Adrenoceptors	α1	Midodrine (agonist)	Increased PPARδ, p-AMPK, and PGC-1α expression in rats	Lee, Kim et al. (2020)
			Reduced lipid content in 3T3-L1 adipocytes	
	α2	Yohimbine (antagonist)	Reversed the noradrenaline induced ERK phosphorylation in sASC of OA patients	Bagdadi et al. (2019)
		Clonidine (agonist)	Decreased BAT thermogenesis and emotional hyperthermia in rats	Antipov, Brizuela et al. (2020)
Adenosine receptors	Non-selective	NECA (agonist)	Reduction in body weight and adiposity in mice	DeOliveira et al. (2017)
	A1	N6-cyclopentyladenosine (agonist)	Reduction in basal lipolysis in mice	DeOliveira et al. (2017)
		Phenylisopropyladenosine (agonist)	Anti-lipolytic effect in human, rat and dog adipocytes	Hoffman et al. (1984), Strong et al. (1993), Leiva, Guzmán-Gutiérrez et al. (2017)
		2-chloroadenosine (agonist)	Anti-lipolytic effect in rats	Johansson et al. (2007), Leiva, Guzmán-Gutiérrez et al. (2017)
		GR79236 (agonist)	Reduction in circulating free fatty acid in rats	Strong et al. (1993), Tozzi and Novak (2017a)
			Promote insulin sensitivity in rats	Meriño et al. (2017), Tozzi and Novak (2017a)
	A2A	CGS21680 (agonist)	Increase in UCP1, PPARγ*, *PRDM16*,* and CIDEA in mice	Gnad et al. (2014), Ruan et al. (2018), DeOliveira et al. (2017)
			Enhanced lipolysis, FDG uptake, glucose homeostasis with increased insulin sensitivity in mice	
		KW60020 (antagonist)	Inhibit thermogenic gene expression in brown adipocyte	Ruan et al. (2018)
		SCH58261 (antagonist)	Increase in fat mass in high-sucurose diet fed rat	Sacramento et al. (2020)
	A2B	BAY 60–6583 (agonist)	Reduced level of aging and oxidative stress markers in mice	Gnad et al. (2020)
		PSB603 (antagonist)	Reduction in oxygen consumption in mice	Gnad et al. (2020)
		MRS1754 (antagonist)	Increase in weight gain in rats	Sacramento et al. (2020)
Lysophospholipid receptors	LPA4-5	Octadecenyl phosphate (agonist)	LPA4 selectively coupling to Gα12/13 proteins in C3H10T1/2 derived adipocytes	Yanagida et al. (2018)
	LPA1–3	Ki16425 (antagonist)		
	S1PR2	JTE013 (antagonist)	UCP1 expression in differentiated primary adipogenic progenitor cells	‘Gohlke, Zagoriy et al. (2019)

### Beta adrenoceptors

**Fig. 2:**
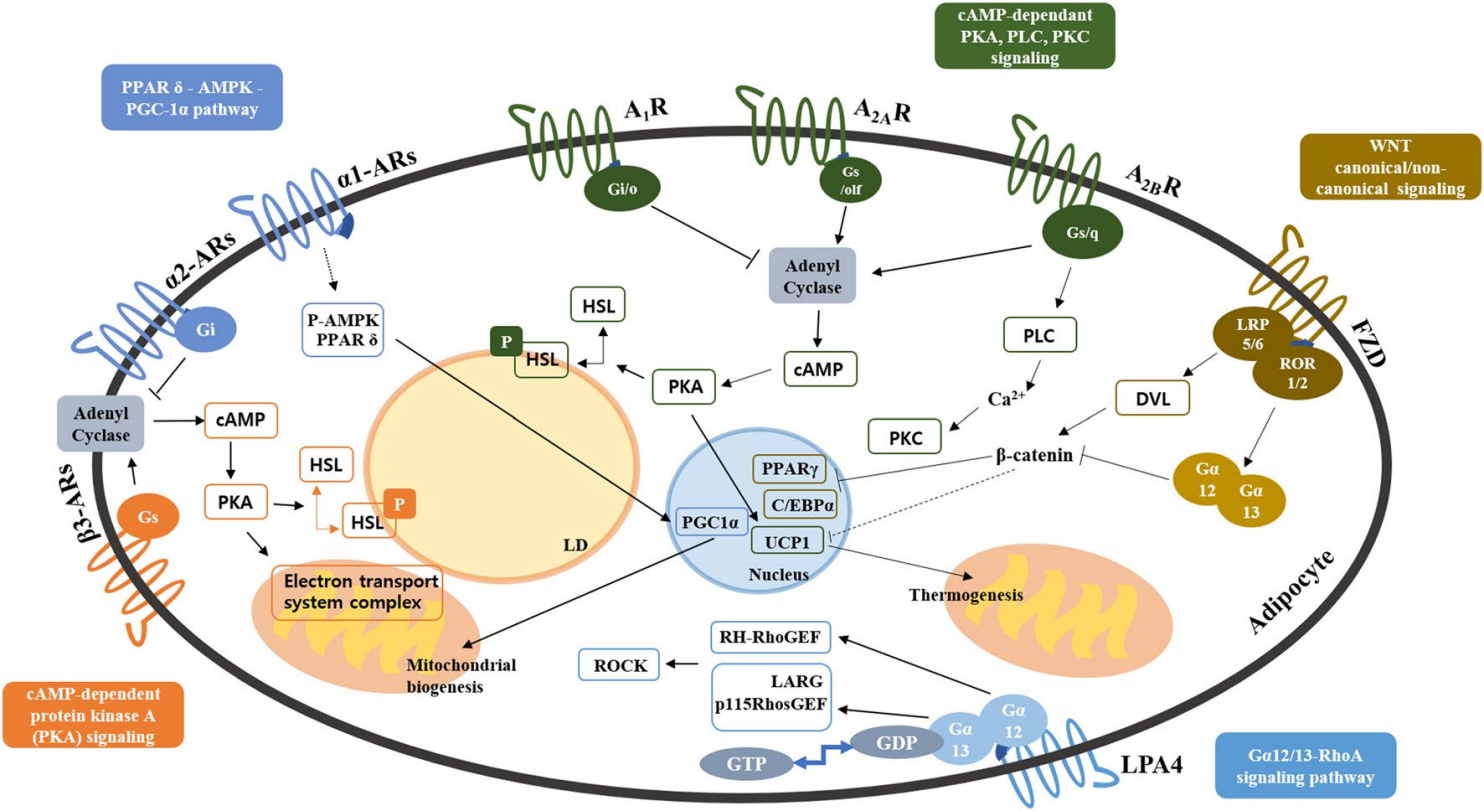
Major signaling pathways of GPCR subfamilies. Figure 1 Metabolic signaling pathways of 5 subfamilies of GPCR: beta adrenoceptors, alpha adrenoceptors, adenosine receptors, frizzled receptors, and lysophospholipid receptors. In adipocytes, activated β3-AR induces cAMP production, and cAMP binding leads to phosphorylation of PKA downstream substrates, such as hormone sensitive lipase (HSL) for lipolysis. The electron transport system complex modulates the oxidative phosphorylation of mitochondria. A_1_R inhibit adenylate cyclase activity and thus decrease cAMP levels, suppressing PKA signaling. A_2A_R activates adenylate cyclase activity, leading to transcription of thermogenic genes like UCP1. Similarly, A_2B_R increases cAMP levels by activation of adenylyl cyclase. A_2B_R also stimulates phospholipase C; thus, both PKA and PKC signaling is promoted. FZD with LRP5/6 cause canonical WNT signaling, which increases the level of β-catenin. This causes inhibition of transcription of adipogenic genes, including PPARγ and C/EBPα. With ROR1/2, FZD causes the reverse effects. WNT signaling is also involved in the expression of thermogenic genes in adipocytes, but the exact pathway is unknown. Upon ligand binding, Gα_12/13_ is activated by the transformation of GDP-bound form to GTP-bound form. Gα_12_ regulates RH-RhoGEF, and Gα_13_ directly stimulates p115RhosGEF and leukemia-associated Rho-GEF (LARG). Rho kinase (ROCK) is a downstream effector of the Gα_12/13_-RhoA signaling pathway

There are three subtypes of beta adrenoceptors (β-ARs), adrenoceptor β1- (β1-AR), β2- (β2-AR), and β3- (β3-AR), expressed in both BAT and WAT (Collins 2012; Hayward, Mueller, and Hasser 2004). β-ARs are coupled to G_s_ proteins and activate adenylyl cyclase that increases intracellular cyclic adenosine monophosphate (cAMP) levels and phosphorylation of protein kinase A (PKA) (Evans et al. 2019) as shown in Fig. 2. Catecholamine-mediated sympathetic activation through β-ARs is a major canonical pathway that triggers lipolysis in brown and white adipocytes (Green et al. 1992; Enocksson et al. 1995; Collins 2012).

Among the three beta adrenoceptors, β3-AR is the most recently discovered subtype and is mainly expressed in adipose tissue (Collins 2012). In adipocytes, activated β3-AR induces cAMP production, and cAMP-dependent PKA phosphorylates downstream substrates, such as hormone-sensitive lipase for lipolysis (Shin et al. 2016; Finlin et al. 2018). Another example of PKA phosphorylation substrates is the electron transport system complex, which modulates mitochondria oxidative phosphorylation (Amer and Hebert-Chatelain 2018). Isoproterenol also induces mitochondrial activity by stimulating β-ARs in adipocytes (Cho et al. 2009). Selective β3-AR agonists have been developed for adipocyte-specific lipolysis activators and anti-obesity therapeutics (Himms-Hagen et al. 1994; Arch 2002). However, clinical trials were unsuccessful due to relatively poor activity in humans compared to preclinical studies (Christopher et al. 2001). It was believed that human white adipocytes lack β3-AR, hindering the anti-obesity effects of β3-AR agonists (Ramseyer and Granneman 2016). However, in more recent studies, it has been reported that human visceral and subcutaneous adipose tissue expresses β3-AR (Ramseyer and Granneman 2016). Along with the recent re-discovery of the metabolically active BAT in adult humans (Cypess et al. 2009; Virtanen et al. 2009), the anti-obesity effects of β3-AR agonists have been revisited in clinical trials. For instance, clinical trials demonstrated that a high dose (200 mg/day) of mirabegron improves BAT thermogenesis and WAT lipolysis, resulting in weight loss (Cypess et al. 2015). Mirabegron is a first-generation selective β3-AR agonist approved for overactive bladder syndrome with a daily dose of 50 mg; thus, the effects of high mirabegron doses on human adipose tissue might be mediated through β1-AR and not by selective activation of β3-AR, as evidenced by the off-target effects, such as tachycardia, reported in these clinical trials (Malik et al. 2012). Several recent clinical studies with a lower dose (50 mg/day) demonstrated that in obese people, mirabegron treatment (10–12 weeks) improved insulin resistance, reduced hemoglobin A1c levels, and increased the protein expression of UCP1 in subcutaneous WAT via cAMP-dependent PKA signaling (Finlin et al. 2018), suggesting the therapeutic potential of the β3-AR agonist.

Although β1-AR and β3-AR are both activated by the same endogenous ligand, norepinephrine, mature brown adipocytes in rodents mainly express β3-AR (approximately 50-fold higher than β1-AR) (Collins et al. 1994) while species differences between humans and rodents should be accounted for (Granneman, Lahners, and Chaudhry 1991). While expression levels of β1‐AR decline during adipogenesis of mouse brown preadipocytes (Bronnikov et al. 1999), β1‐AR activation increases cAMP levels and facilitates cell proliferation of mouse brown preadipocytes *in vitro* (Bronnikov, Houstĕk, and Nedergaard 1992). Consistently, β1-AR is required for cold exposure-induced progenitor proliferation in mouse BAT (Lee et al. 2015). Although the predominant subtype is β3-AR, β1-AR mediates cold-induced thermogenesis of BAT in mice (Ueta et al. 2012) and has compensatory effects in BAT of β3-AR-deficient mice (Chernogubova et al. 2005a). Similarly, *in vitro* β1-AR knockdown in immortalized human brown adipocytes reduced UCP1 induction following isoproterenol treatment (Evans et al. 2019; Riis-Vestergaard et al. 2020). Treatment with talinolol, a β1-AR selective antagonist, reduces cold exposure-induced browning in mice (Jiang, Berry, and Graff 2017).

Although β2-AR can be found in BAT (Levin and Sullivan 1986; Chernogubova et al. 2005b; Rothwell, Stock, and Sudera 1985), it is thought to be expressed mostly in the blood vessels of rodent BAT (Bengtsson, Cannon, and Nedergaard 2000; Chernogubova et al. 2005b). Indeed, β2-AR stimulation increases blood flow and metabolic activity of BAT, without direct effects on oxygen consumption rates of isolated adipocytes (Ernande et al. 2016).

### Alpha adrenoceptors

There are two types of alpha adrenoceptors, α1 and α2, each with three subtypes, namely, α1A, α1B, α1D and α2A, α2B, α2C (Evans et al. 2019). As described in Fig. 1, α1 adrenoceptors (α1-AR) act on G_q/11_ proteins to activate phospholipase C, while α2-ARs modulate Gi/o regulatory proteins to inhibit adenylyl cyclase (Evans et al. 2019). In a study on synovial adipose tissue-derived mesenchymal stem cells (ASCs), α2-ARs were found to be highly expressed in monolayer ASCs, compared to α1-AR (El Bagdadi et al. 2019).

Pharmacological activation of α1-AR in differentiated 3T3-L1 adipocytes by midodrine induces mitochondrial oxidative phosphorylation and ATP production through a PPARδ-AMPK-PCG-1α pathway (Lee et al. 2020). Midodrine upregulates PPARδ, p-AMPK, and PGC-1α protein levels, and reduces lipid content in differentiated 3T3-L1 adipocytes (Lee et al. 2020). These consequences were reversed by GSK0660, a PPARδ antagonist (Lee et al. 2020).

In adipocytes, α2-AR activation inhibits lipolysis since it is negatively coupled to adenylyl cyclase (Lafontan and Berlan 1981). A study using transgenic knockout mice suggested a possible association between α2-AR and adipocyte hyperplasia since mice expressing α2-ARs in fat, in the absence of β3-ARs (β3-AR -/- background), develop high-

fat diet-induced obesity (Valet et al. 2000). Although BAT is not significantly affected by transgenic expression (Valet et al. 2000), clonidine, an α2-AR agonist, reduces BAT thermogenesis (Antipov et al. 2020).

### Adenosine receptors

Adenosine exerts a variety of effects in adipose tissue via multiple receptor subtypes, including A_1_, A_2A_, A_2B_, and A_3_ (Leiva et al. 2017). They couple to different G proteins, stimulating or inhibiting adenylyl cyclase. For instance, A_1_ receptor (A_1_R) and A_3_R couple to G_i/o_ proteins, causing inhibition of cAMP production by adenylyl cyclase and downregulation of PKA signaling, while A_2A_R and A_2B_R couple to G_s/olf_ proteins, promoting cAMP-mediated signaling pathways (Tozzi and Novak 2017b). These receptors are known to be associated with adipogenesis, lipolysis, adipose tissue inflammation, and insulin resistance (Csóka et al. 2014; Eisenstein et al. 2014), implying their potential as a pharmaceutical target for obesity and related metabolic syndromes (Pardo et al. 2017).

A_1_Rs are highly expressed in human white adipocytes, and when activated, they inhibit adenylate cyclase activity and cAMP production, leading to WAT lipolysis inhibition (Antonioli et al. 2015; Gnad et al. 2014). A reduction in the *ex vivo* basal lipolysis rate was observed in obese mice treated with N^6^-cyclopentyladenosine (CPA), an A_1_R agonist (DeOliveira et al. 2017). In addition, plasma levels of non-esterified fatty acids, glycerol, and triglycerides were reduced in CPA-treated wild-type mice (Johansson et al. 2008). Other A_1_R agonists, such as phenylisopropyladenosine, GR79236, and 2-chloroadenosine, exhibit anti-lipolytic effects following lipolysis induction in adipocytes from humans, rats, transgenic mice, and dogs (Hoffman et al. 1984; Strong et al. 1993; Johansson et al. 2007; Tozzi and Novak 2017a; Leiva et al. 2017). GR79236 also promotes insulin sensitivity in adipose tissue through a reduction in circulating FFA and triglyceride levels (Meriño et al. 2017). Simultaneously, A_1_R signaling promotes lipogenesis and modulates inflammation, shown by the absence of visceral adipose tissue (VAT) accumulation and proinflammatory cytokines (IL-1β, IL-6, IL-12, and TNF-α) in A_1_R knockout mice (Yang et al. 2015).

In contrast to A_1_R, A_2A_ receptors (A_2A_R) have a significantly higher expression in human brown adipocytes than in white adipocytes (Gnad et al. 2014), and stimulate adenylate cyclase activity to promote lipolysis (Antonioli et al. 2015). It has also been reported that A_2A_R signaling is necessary for the complete physiological functions of BAT (Gnad et al. 2014). Expression of thermogenic genes, including UCP1, PPARγ*, *PRDM16*,* and CIDEA, is significantly increased by CGS21680, an A_2A_R agonist, both *in vitro* and *in vivo* (Gnad et al. 2014; Ruan et al. 2018). Meanwhile, KW60020, an A_2A_R antagonist, inhibits thermogenic gene expression (Ruan et al. 2018).

Enhancement in oxygen consumption, lipolysis, and fluorodeoxyglucose uptake was observed in mice injected with CGS21680 (Gnad et al. 2014). This improvement in glucose homeostasis correlating with increased insulin sensitivity was observed in obese mice treated for two weeks with CGS21680 (DeOliveira et al. 2017). Additionally, a high dose (0.05 mg/kg) of 5-N-ethylcarboxamidoadenosine, a non-selective agonist, resulted in a significant reduction in body weight and adiposity in obese mice. Consistently, A_2A_R knockout mice gained more weight and showed an increase in visceral fat mass and adiposity after high-fat feeding (Ya et al. 2018). Chronic administration of 0.5 mg/kg SCH58261, an A_2A_R antagonist, in rats fed with a high-sucrose diet, led to an increase in fat (Sacramento et al. 2020). On the contrary, disruption of A_2A_R resulted in the aggravation of a high-fat diet-induced adipose tissue inflammation (Ya et al. 2018).

A_2B_ receptor (A_2B_R) is abundantly expressed in human BAT and is involved in its activity (Gnad et al. 2020). Pharmacological stimulation of A_2B_R causes a significant increase in oxygen consumption, while administration of PSB604, an A_2B_R antagonist, or inhibition of A_2B_R, have the opposite effects (Gnad et al. 2020). In addition, a protective effect against age-induced oxidative stress was investigated in wild-type mice treated with BAY 60–6583, an A_2B_R agonist (Gnad et al. 2020). This A_2B_R stimulation increased the expression of thermogenic markers such as UCP1 in human WAT (Gnad et al. 2020). Conversely, the A_2B_R antagonist MRS1754, promoted a significant weight gain in female and male rats (Sacramento et al. 2020).

Contradictory results from previous studies showed that adenosine inhibited the stimulatory effects of isoproterenol on oxygen uptake, lipolysis, and respiration in brown adipocytes of hamsters (Szillat and Bukowiecki 1983; Schimmel and McCarthy 1984). However, these discrepancies could be due to species-specific differences in the expression levels of adenosine receptor isotypes (Gnad et al. 2014).

As mentioned earlier, along with A_1_R, the A_3_ receptor (A_3_R) is also inhibitory since it couples with inhibitory Gi/Go proteins to decrease cAMP levels (Pardo et al. 2017). Although both BAT and WAT express A_3_R (Gnad et al. 2014), its activity in adipose tissue has not been thoroughly studied (DeOliveira et al. 2017). For instance, pharmacological inhibition of A_3_R with MRS1523 did not show significant lipolytic effects in murine brown adipocytes but it might indirectly contribute to metabolic activity, such as glucose homeostasis, through liver involvement (Gnad et al. 2014; Pardo et al. 2017).

### Frizzled receptors

Frizzled (FZD) receptors, comprised of 10 subtypes (FZD1-FZD10) (Zeng, Chen, and Fu 2018), are the main receptors for WNT ligands (Wang et al. 2016). They are considered unconventional GPCR proteins due to the lack of evidence of their interactions with G proteins (Nichols et al. 2013). However, recent studies have discovered the involvement of heterotrimeric G proteins in WNT/FZD signaling (Schulte and Wright 2018). Although FZD receptors are studied mainly through WNT signaling (Dijksterhuis, Petersen, and Schulte 2014; Petersen et al. 2017), understanding the GPCR nature of FZD receptors would enable their targeting to treat diseases (Schulte and Wright 2018). As WNT receptors, FZD receptors can act via three distinct pathways: canonical/β-catenin, non-canonical/non-β-catenin, and the WNT and Ca^2+^ pathways (Nakamura et al. 2016). FZD genes, including FZD1, 2, 3, and 7, are highly expressed in VAT (Zuriaga et al. 2017).

In adipose tissue, it is known that FZD receptor-mediated WNT signaling affects adipogenesis and depending on the WNT type, it can promote or inhibit adipogenesis (van Tienen et al. 2009). For example, β-catenin-dependent WNT genes, including WNT10b and WNT3a, have been reported to inhibit adipogenesis by inhibiting PPARγ and C/EBPα expression (Nishizuka et al. 2008) while β-catenin-independent WNTs, including WNT5a and WNT5b, exert the opposite effect (Park et al. 2015). It has also been reported that WNT ligands can antagonize the function of one another as alternative WNT-YAP/TAZ-TEAD signaling, which couples to Gα_12/13_ proteins, activated by WNT5a, can suppress the effects of WNT/β-catenin signaling that restrain adipogenesis (Park et al. 2015). Moreover, it has been shown that WNT3a promotes oxygen consumption and mitochondrial gene expression in ear mesenchymal stem cells adipocytes (Mori et al. 2012). FZD receptors can function differently depending on their ligands and co-receptors. For example, low-density lipoprotein receptor-related protein 5 (LRP5) or LRP6 activates the β-catenin signaling pathway, which inhibits adipocyte differentiation, while the FZD1 receptor and ROR1/2 co-receptor interacting with WNT5a/b can promote adipogenesis and inhibit the WNT-FZD/LRP pathway (Park et al. 2015; Loh et al. 2015). Metabolic stimulation, such as a high-fat diet, also affects WNT/β-catenin signaling, causing activation of VAT adipocyte precursors “over-proliferation,” which results in depot-specific hypertrophy and hyperplasia (Chen and Wang 2018). In addition, it has been reported that WNT signaling is associated with adipocyte browning, as pharmacological and genetic inhibition of WNT signaling resulted in an increased expression of thermogenic markers (UCP1, CIDEA) in primary mouse adipocytes (Lo et al. 2016). Recent studies have reported the possible WNT-FZD involvement in beige adipocytes differentiation (Chen and Wang 2018).

### Lysophospholipid receptors

Lysophospholipids are bioactive lipid molecules that activate their specific GPCR as extracellular mediators (zu Heringdorf 2008). They are essential for cell growth and death and act as signaling molecules at inflammatory sites (Cas et al. 2020). Obesity impairing lysophospholipid metabolism generates inflammation and insulin resistance (Del Bas et al. 2016). Patients with type 1 diabetes and murine plasma lipid profiles were found to have decreased levels of long-chain lysophospholipids (Cas et al. 2020).

Lysophospholipid receptors, widely expressed in mammals, respond to lysophosphatidic acid (LPA) and sphingosine-1-phosphate (S1P) (Lemos et al. 2018). LPA and S1P receptors couple to different G protein types (Table 2) and are emerging as important pharmacological targets with a research potential (Yanagida et al. 2018).

LPA is highly responsive to the control, traffic, and activation of immune cells and is therefore strongly associated with inflammatory diseases such as obesity and diabetes (Lemos et al. 2018). There are six types of LPA receptors, named LAP1-6 (Yung, Stoddard, and Chun 2014). An *in vitro* study showed that LPA1, LPA4, and LPA6 are expressed in C3H10T1/2 derived adipocytes (Yanagida et al. 2018). Studies using octadecenyl phosphate, a LPA4/5 agonist, and Ki16425, a LPA1–3 antagonist showed that LPA4 predominantly activates Gα_12/13_-RhoA pathway in C3H10T1/2 derived and primary cultured adipocytes (Yanagida et al. 2018). Rho-associated protein kinase (ROCK) is a downstream effector of the Gα12/13-RhoA signaling pathway (Yang et al. 2020), and ROCK inhibitor treatment (Y-27632) and genetic deletion of ROCK enhance adipogenesis of 3T3-L1 and mouse embryonic fibroblast (MEF) cells (Noguchi et al. 2007). Moreover, LPA treatment reduces differentiation of mouse primary brown preadipocytes, and overexpression of LPA-generating enzyme autotaxin (ATX) in mice significantly reduces expression levels of brown adipocyte markers and increases susceptibility to diet-induced obesity partly by limiting brown adipogenesis (Federico et al. 2012).

An *in vivo* study of LPA4 knockout mice showed upregulated mitochondrial and adipogenic gene expression in WAT (Yanagida et al. 2018). LPA4 limits the healthy expansion of WAT via Gα_12/13_ proteins in adipocytes; thus, a diet-induced obesity mouse model of LPA4 abrogation resulted in metabolically healthy obese phenotypes with ameliorated WAT inflammation and insulin resistance (Yanagida et al. 2018).

S1P receptors (S1PR) have five subtypes, namely S1PR1-5 (Hla and Brinkmann 2011). In adipose tissue, S1PR1, 4, and 5 are highly expressed in both BAT and WAT, while S1PR2 and S1PR3 are equally expressed in adipogenic progenitor cells (APCs) and its whole tissue (Gohlke et al. 2019). They couple to various G proteins; for instance, S1PR1 couples to Gα_i_, S1PR2 to Gα_s_, Gα_q_, or Gα_12/13_, S1PR3-5 to Gα_i_, or Gα_12/13_ (Table 2).

S1P concentrations are increased in adipose tissue of obese people (Ito et al. 2013). Similarly, S1P attenuated norepinephrine-induced expression of brown adipogenic genes in APCs obtained from mouse BAT (Gohlke et al. 2019). An *in vitro* study of primary mouse APCs was conducted with S1P receptor-specific antagonists: VPC23019 for S1PR1 and 3, JTE013 for S1PR2, and CYM50358 for S1PR4; it was found that S1PR2 was the most relevant subtype involved in adipocyte browning due to its particularly high expression in ASCs and increase in UCP1 expression induced by S1PR2 inhibition (Gohlke et al. 2019). In addition, S1PR2 mRNA expression levels were lower in mature adipocytes compared to APCs of mouse BAT and WAT (Gohlke et al. 2019).

## Discussion

Metabolic dysfunction of adipose tissue has been investigated as a key pathogenic mechanism leading to obesity-related metabolic diseases (Rosen and Spiegelman 2014). Considering the critical roles that adipocytes have in metabolic homeostasis (Rosen and Spiegelman 2006), adipose tissue remains a promising target for new metabolic diseases therapeutics to restore energy balance and immune/endocrine function. With recent advances in our understanding of the molecular mechanisms regulating the development and activation of BAT, multiple endogenous or pharmacological activators have been tested to induce BAT activation/expansion and WAT browning in humans (Bonet, Oliver, and Palou 2013; Kiefer 2017). Although there are currently no drug candidates demonstrating successful anti-obesity effects in clinical trials, many novel targets that regulate thermogenic pathways in adipose tissue have been identified in recent years (Loh, Kingwell, and Carey 2017). GPCRs are one of the most intensively investigated drug target families, and their broad disease indications might provide opportunities to develop novel therapeutics for metabolic diseases. In addition, there is a wealth of structural information and established functional assays for GPCR regulators. Therefore, understanding the roles of GPCRs in adipose tissue metabolism could help identify hidden disease–drug relationships and accelerate the drug discovery process for obesity and metabolic disorders.
